# Electroencephalogram monitoring during ketamine antidepressant treatment: a pilot study

**DOI:** 10.1192/j.eurpsy.2024.778

**Published:** 2024-08-27

**Authors:** M. S. Fabus, D. Casey, K. Warnaby, M. Woolrich, R. McShane

**Affiliations:** ^1^Nuffield Department of Clinical Neurosciences, University of Oxford; ^2^Interventional Psychiatry, Oxford Health NHS Foundation Trust; ^3^Department of Psychiatry, University of Oxford, Oxford, United Kingdom

## Abstract

**Introduction:**

Depression is a major cause of disability world-wide. Up to a third of patients have a treatment-resistant form (TRD), presenting a major challenge. Ketamine has been introduced as a novel rapid-acting antidepressant effective in this population. However, at present, ketamine treatment is not routinely informed by any objective neural markers. Basic research has shown promising electroencephalogram (EEG) changes including a decrease in alpha power. However, clinical translation is lacking.

**Objectives:**

Assess the feasibility of identifying EEG correlates of ketamine infusions in a routine outpatient setting with a low-cost, easily usable system.

**Methods:**

The study was carried out at the Oxford Health Foundation Trust Ketamine Clinic (ethics reference 22/EM/0226). N=18 EEG recordings from N=12 patients were collected (5 women, mean age 44, range 33-62, IV dose 0.5-1mg/kg over 40min). 4-channel EEG was collected with a Muse-S headband at 256Hz, from 5min before to 55min after infusion start. 5s epochs were rejected if gyroscope data indicated head movement above 10 deg/s or if amplitude was above 200μV. A spectrogram (4s window, 3s overlap) as well as band-limited power (theta: 4-8Hz, alpha: 8-13Hz, beta: 13-25Hz) were computed. Significance of changes was found with a repeated measures analysis of variance (RM-ANOVA) on power in 5min segments together with post-hoc Tukey’s P-values.

**Results:**

Across the ketamine infusion recordings, there was a significant effect of time (F=3.65, P=0.0105) and Channel*Time interaction (F=3.80, P<0.001) on the EEG spectrum. Effects were largest on temporal electrodes, particularly TP9 in the alpha and theta bands (Figure 1, Table 1).

**Table 1:** Effect sizes (Cohen’s d) and FDR-corrected ANOVA P-values for ketamine effects on each EEG channel and frequency band. P<0.05 was considered significant (bold). n.s. = not significant (P>0.2).

Channel / Band	TP9	AF7	AF8	TP10
Theta	**1.16 (P=0.019)**	0.11 (n.s.)	0.11 (n.s.)	0.42 (P=0.113)
Alpha	**1.41 (P<0.001)**	0.12 (n.s.)	0.17 (n.s.)	0.605 (P=0.113)
Beta	1.19 (P=0.112)	0.03 (n.s.)	0.08 (n.s.)	0.21 (n.s.)

**Image:**

**Figure:**
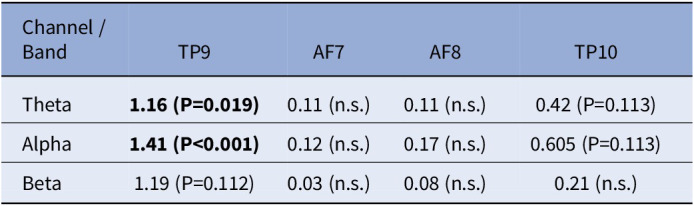

**Conclusions:**

In a routine outpatient setting, sub-anaesthetic ketamine infusions in TRD patients were associated with decreased fronto-temporal EEG alpha and theta power. Future work should assess the potential of low-cost routine EEG, and alpha desaturation specifically, to inform individualised ketamine treatment.

**Disclosure of Interest:**

None Declared

